# Mutators Enhance Adaptive Micro-Evolution in Pathogenic Microbes

**DOI:** 10.3390/microorganisms10020442

**Published:** 2022-02-15

**Authors:** Kylie J. Boyce

**Affiliations:** School of Science, RMIT University, Melbourne, VIC 3001, Australia; kylie.boyce@rmit.edu.au; Tel.: +61-(0)3-9925-7101

**Keywords:** mutator, mismatch repair, mutS, msh2, adaptation, micro-evolution, antibiotic resistance, pathogen, phenotypic diversity, genotypic diversity

## Abstract

Adaptation to the changing environmental conditions experienced within a host requires genetic diversity within a microbial population. Genetic diversity arises from mutations which occur due to DNA damage from exposure to exogenous environmental stresses or generated endogenously through respiration or DNA replication errors. As mutations can be deleterious, a delicate balance must be obtained between generating enough mutations for micro-evolution to occur while maintaining fitness and genomic integrity. Pathogenic microorganisms can actively modify their mutation rate to enhance adaptive micro-evolution by increasing expression of error-prone DNA polymerases or by mutating or decreasing expression of genes required for DNA repair. Strains which exhibit an elevated mutation rate are termed mutators. Mutators are found in varying prevalence in clinical populations where large-effect beneficial mutations enhance survival and are predominately caused by defects in the DNA mismatch repair (MMR) pathway. Mutators can facilitate the emergence of antibiotic resistance, allow phenotypic modifications to prevent recognition and destruction by the host immune system and enable switching to metabolic and cellular morphologies better able to survive in the given environment. This review will focus on recent advances in understanding the phenotypic and genotypic changes occurring in MMR mutators in both prokaryotic and eukaryotic pathogens.

## 1. Introduction

**Figure 1:**
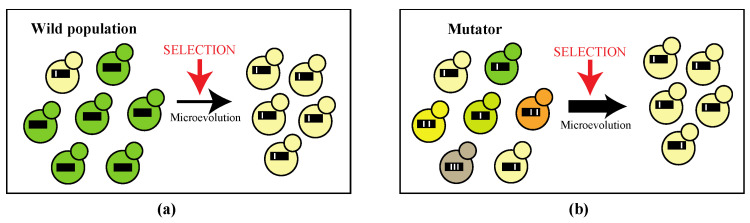
(**a**) In wild microbial populations, the rate of mutations is kept low in favor of long-term genetic stability. Upon changing conditions within the host natural selection ensues cells possessing beneficial mutations (cream-colored cell) become predominant in the population in a short time frame in the process of micro-evolution; (**b**) Micro-evolution is enhanced in populations containing mutators, cells which possess an elevated mutation rate, as there is higher genetic diversity within a population on which selection can act. Black lines represent the microbial genome with white bars as mutations. Different phenotypes are indicated by colors.

The evolutionary success of pathogenic microorganisms is governed by their capacity to deal with environmental stress and rapidly adapt to the changing conditions experienced within the host. Natural selection ensures cells possessing beneficial mutations become predominant in the population in a short time frame in a process termed adaptive micro-evolution (Figure 1). Adaptive micro-evolution is greatly enhanced by an increased mutation rate, which provides higher genetic diversity within a population on which selection can act [1,2,3].

Upon exposure to stress, such as that experienced in the host environment, microorganisms can increase the mutation rate through two mechanisms: stress-associated mutagenesis and stress-induced mutagenesis. Stress-associated mutagenesis refers to the mutations which arise due to exposure to environmental stress such as the unavoidable chemical processes occurring continuously in cells, by the generation of oxygen radicals by cells of the host’s immune system during infection or exposure to other mutagens which damage DNA. Stress-induced mutagenesis occurs when cells actively modify the mutation rate through increasing expression of error-prone DNA polymerases or by mutating or decreasing expression of genes required for DNA repair [4]. These mutagenic processes can be linked. For example, in bacteria, the SOS global response to environmental DNA damage initially activates a high-fidelity DNA repair pathway but will sequentially activate mutagenic repair if the damage is insufficiently repaired. Strains which exhibit an elevated mutation rate, often 100–200-fold that of wildtype, are termed mutators. Although a mutator phenotype is expected to ultimately reduce fitness, a high prevalence of mutators is observed in clinical populations of pathogenic microbes, suggesting this phenotype is advantageous in the host environment. Mutators are associated with enhanced evolution of resistance to antibiotics, avoiding recognition by cells of the host’s immune system and adaptation to specific environmental niches within the host [5,6]. In bacteria, a mutator phenotype can be generated by defects in proofreading of the epsilon sub-unit of DNA Polymerase III, lack of DNA Polymerase I, defective DNA mismatch repair, a lack of GATC hemimethylation, a reduction in the neutralization of reactive oxygen species, defects in the base excision repair of oxidative damage or deaminated cytosine and alterations to tRNAs [7,8]. Mutators in eukaryotic cells arise from defects in mismatch repair, base excision repair, nucleotide excision repair and double strand break repair. A mutator phenotype can also be generated from mutations in genes encoding the DNA Polymerase delta sub-unit, DNA damage checkpoint proteins, DNA damage response signaling proteins, DNA damage tolerance proteins, proteins involved in trans-lesion synthesis during post-replication repair, histone and chromatin assembly regulators, proteins which promote error-free DNA repair and tolerance to oxidative stress. However, mutators found in clinical populations are predominately in the DNA mismatch repair (MMR) pathway [6,9]. This review will focus on recent advances in understanding the phenotypic and genotypic changes occurring in MMR mutators in both prokaryotic and eukaryotic pathogens by collating information from recent publications.

## 2. Repair of DNA Replication Errors: DNA Polymerase and Mismatch Repair (MMR)

The mechanisms for replicating DNA prior to cell division are extremely accurate to ensure genetic stability. Errors generated during DNA replication are corrected by the action of two sequential repair systems, DNA polymerase 3′–5′exonuclease activity and the MMR pathway, which multiplicatively increase replication fidelity to a final error rate of 10^−10^ per bp replicated [10]. In bacteria such as *Escherichia coli*, replication errors are corrected by the 3′–5′ editing exonuclease activity of the ε sub-unit of DNA polymerase III and the MMR pathway comprised of *mutS*, *mutL*, *mutH* and *uvrD*. A MutS homodimer recognizes mismatched bases, binds at the mismatch and is detected by a MutL homodimer. The MutS–MutL–DNA complex activates MutH, and, in the *E. coli* model, initiates base removal by nicking the newly synthesized unmethylated strand. The UvrD helicase acts with one of the four exonucleases (ExoI, Exo VII, ExoX or RecJ) to perform the excision from the strand break. Loss of exonuclease activity in the *E. coli* DNA polymerase III ε sub-unit is lethal as it gives rise to a 1000–10,000-fold increase in mutation rate which exceeds the error threshold for haploid cells (one inactivating mutation per essential gene per cell division, which corresponds to ~1000 fold increase in the wildtype mutation rate) [11,12]. Defects in the MMR pathway in bacteria lead to a ~100-fold increase in mutation rate and a subsequent mutator phenotype, in which the mutation rate is elevated [5,13,14]. MMR also prevents recombination between divergent sequences, so mutator strains are also hyper-recombinogenic, and this facilitates the acquisition of DNA by horizontal transfer [10,15,16].

Bacteria can respond to stress by actively elevating their mutation rate by increasing expression of error-prone DNA polymerases or decreasing expression of genes of the MMR pathway [4]. For example, expression of *mutS* has been shown to decrease in some *E. coli* strains during the transition to stationary phase growth, and expression of *mutS* is also decreased in *E. coli*, *Pseudomonas aeruginosa* and *Vibrio cholerae* in response to antibiotics [17,18]. Another mechanism to achieve an increased mutation rate is through incision or excision of mobile elements into MMR genes. In response to the environmental stress of entering stationary phase, a prophage integrated between *mutS* and *mutL* in strains of *Streptococcus pyogenes* causes the transcription of *mutL* to be halted, leading to a temporary and reversible 100-fold increase in the mutation rate [19]. In the marine bacterium, *Vibrio splendidus*, excision of a mobile element residing in *mutS* has been shown to increase mutation rate [20]. When the mobile element is integrated at *mutS*, expression is driven by the mobile element promoter to produce a functional MutS protein. However, upon mobile element excision, the *mutS* promoter is re-joined to the native *mutS* promoter, leaving a 2 bp *mutS* deletion and a consequent frameshift mutation which results in no functional MutS produced and a mutator phenotype [20]. Hundreds of bacteria in *Betaproteobacteria* and *Gammaproteobacteria* possess similar mobile elements in *mutS* but leave an intact *mutS* after excision [20]. Some bacteria lineages which have co-evolved with their animal hosts have completely lost genes involved in MMR. Species of *Spiroplasma*, which are plant, *Drosophila* and animal endosymbionts, lack the MMR genes *mutS* and *mutL* and have faster evolution in comparison to other insect symbionts [21]. Analysis of genomic evolution of *Spiroplasma poulsonii* over several years both within *Drosophila* and in vitro revealed substitution rates higher than reported in other bacteria and the dynamic evolution of toxins which result in *Spiroplasma*-induced phenotypes in the host fly [21]. The genome of *Helicobacter pylori*, a bacteria with a long association with humans, lacks the MMR genes *mutS*, *mutL* and *mutH* and exhibits mutation rates 10–100 times higher than those for *E. coli* [22]. Recent evidence suggests *H. pylori* can also actively regulate mutation rates, as these differ between sub-populations of cells [23]. Slipped-strand mispairing of a homonucleotide tract in the *mutY* base excision repair gene leads to frameshifts that eliminate gene function and result in a further ~26-fold increase in mutation rate compared to wildtype [23].

The sequential repair system is structurally and functionally conserved in eukaryotes (reviewed in [24,25]). The Pol2 (ε) and Pol3 (∂) DNA polymerases, which primarily replicate the leading and lagging strands, respectively, also have 3′–5′ exonuclease activity, and sequential repair is performed by the analogous MMR pathway. However, eukaryotic MMR requires a pre-existing nick instead of hemimethylation as the strand discrimination signal and has multiple MutS and MutL homologs which work as heterodimers. MMR in eukaryotic microbes is best characterized in *Saccharomyces cerevisiae*, in which the genome encodes six MutS homologs (*MSH1-6*) and four MutL homologs (*MLH1-3* and *PMS1*). Only *MSH2*, *MSH3*, *MSH6*, *MLH1* and *PMS1* are required for mismatch repair of nuclear DNA, with *MSH3* and *MSH6* playing partially redundant roles [25]. In eukaryotes, the MutS heterodimers are referred to as MutSα(Msh2–Msh6) and MutSβ (Msh2–Msh3) and are redundant with respect to repairing small insertions and deletions (indels) but are specialized to remove specific base–base mismatches and large insertion–deletion loops, respectively. The MutSα/ß heterodimer is tethered to a DNA clamp (the proliferating cell nuclear antigen (PCNA) that acts as a processivity factor for Pol3 (DNA polymerase ∂). After mismatch recognition, a MutL heterodimer referred to as MutLα (comprising Mlh1–Pms1) interacts to form a ternary complex that leads to nicks on the new strand and subsequent strand removal and resynthesis. Mutations which abolish exonuclease activity in the Pol2 (ε) and Pol3 (∂) DNA polymerases or MMR activity in Msh2, Mlh1, Pms1 and, to a lesser extent, Msh3 and Msh6, result in a mutator phenotype in which the mutation rate is elevated [24,26,27]. A lack of Pol3 3′–5′ exonuclease activity results in an increased frequency of transitions and transversions [26,27]. MMR mutants result in an increased proportion of transitions and single base pair indels within homopolymeric tracts including di- and tri-nucleotide microsatellites [28,29,30,31,32,33]. The differences in mutational profiles suggest specific roles of each repair component with Pol3 exonuclease activity, and not MMR, repairing the majority of mismatches resulting in transversions and mismatches in homopolymeric tracts occurring from DNA polymerase slippage repaired predominantly by MMR, and not Pol3 [27,28,29]. Loss of Pol2 and Pol3 exonuclease activity results in a ~12- and ~130-fold increase in mutation rate, respectively [26,27]. Defects in eukaryotic MMR result in ~200-fold increase in mutation rate [24,27,29]. Lack of both Pol3 exonuclease activity and MMR is lethal in *S. cerevisiae*, as error-induced extinction occurs within 6–7 mitotic divisions due to a 10,000-fold increase in mutation rate [11,12,27,34]. In contrast, a Pol2 exonuclease activity and MMR double mutant is viable but exhibits poor growth, with mutation rates of ~520-fold higher than the wildtype strain [26]. It should be noted that measuring mutation rates is challenging and has many assumptions and limitations [35]. Estimates can vary depending on the method used; for example, using the gold standard method of the Luria and Delbruck fluctuation assay the *msh2* mutant in *S. cerevisiae* was estimated to have a 40-fold increase in mutation rate compared to wildtype, but whole genome sequencing shows this to be >200-fold [29,35].

A number of recent studies have shown that some fungal species have also lost genes encoding DNA repair genes from their lineages [36,37]. Species in the *Hanseniaspora* bipolar yeast lineage have a reduced number of cell cycle and DNA repair genes and a subsequent increased mutational burden but have higher evolution rates enabling faster adaptation to the environment [36]. A comparison of the genomes of ascomycete yeasts shows that DNA repair genes, including the orthologues of the MMR genes *MLH1-3*, are less conserved than other gene categories [37]. A comparison of 1107 species of ascomycete fungi showed that four closely related obligate plant parasites from the powdery mildew genera (*Erysiphe* and *Blumeria*) have lost a substantial number of MMR genes and exhibit accelerated sequence evolution [38].

## 3. Mutators with Defective MMR Are Prevalent in Clinical Populations of Bacterial Pathogens

In wild bacterial populations, the rate of mutations is kept low in favor of long-term genetic stability [39]. In contrast, clinical populations contain a high proportion of mutators at levels that suggest that having an increased mutation rate is favorable in the host environment [40]. For example, *E. coli* mutators have a selective advantage in urine and during the late stages of infection in a mouse model of urinary tract infection [41]. In vitro experiments have revealed that a mutator phenotype is advantageous in rapidly changing environmental or stressful conditions. Long-term *E. coli* laboratory evolution experiments often evolve mutator phenotypes which can sweep the population and are co-selected with beneficial mutations in clonal populations [3,10,39,42]. Evolution experiments with *E. coli* have shown that mutator strains adapt faster than control populations and become predominant in mixed populations under stressful or changing conditions [39,40,42]. The larger the population size, the more likely the fixation of mutator alleles [40]. When *E.coli* is consigned to selective conditions in which only one particular mutant can grow, the frequency of the mutator phenotype is elevated from 0.001% to 100% within two rounds of consecutive selection [43]. Most mutations are deleterious, and therefore, a mutator phenotype is expected to ultimately reduce fitness. This has been shown in *E. coli* in vitro experiments where bottlenecks, which encourage the accumulation of mutations, result in large losses in fitness and extinction in some cases in mutator strains [44]. Virulence is also often reduced in mutators due to the accumulation of mutations in critical genes [45,46]. Evolution experiments of wildtype and *mutS E. coli* mutators have also shown that the overall fitness of evolved mutator strains can increase under non-adapted growth conditions due to co-selection with beneficial mutations [47].

Bacterial species contain mutators within natural populations at varying levels of prevalence; however, the frequency of mutators is higher in clinical populations compared to environmental populations (Table 1). For example, ~22% of *Stenotrophomonas maltophilia* clinical isolates are mutators compared to 5% of environmental isolates [48]. This trend is thought to come about due to the requirement to adapt to rapidly fluctuating environmental conditions within the host such as limiting nutrients, phagocytic attack from the immune system and changing and extreme selection from anti-microbial therapy. In addition, even within clinical populations a higher prevalence of mutators is observed in specific conditions. For instance, the prevalence of *P. aeruginosa* mutators from chronic infections of cystic fibrosis (CF) patients, whose lung’s possess increased viscosity and osmolarity conditions, is significantly higher (30–60%) than isolates from infections of non-CF patients or those in acute infections [6,49,50,51,52,53]. A higher prevalence of mutators is also exclusively observed in *P. aeruginosa* infections in non-CF bronchiectasis or chronic obstructive pulmonary disease patients [54,55]. The higher prevalence of mutators in chronic infections of CF patients is thought to result from exposure to reactive oxygen species (ROS) released from leukocytes of the host during lung inflammation [52]. *P. aeruginosa* mutators are found after 5 years of chronic infection, and then prevalence increases over time [52]. Once they arise, mutator strains, which have higher tolerance to oxidative damage to DNA, accumulate mutations which confer a selective advantage in the host to allow pathogen persistence [52]. Likewise, a higher incidence of mutators was observed in *Staphylococcus aureus* isolates from CF patients (14.6%) compared to non-CF patients (1.35%) and in *Burkholderia cepacia* complex species in chronically infected CF patients (40.7%) [56,57]. Mutations have been identified mostly in the *mutS* gene as this appears to be the most common genetic cause of a mutator phenotype in most species (Table 1) [54,56,57,58,59,60]. Mutations have also been identified in *mutL*, *mutH* and *uvrD* [50,57,58,59,61,62] (Table 1).

Other genetic factors besides MMR mutations may be contributing to increased mutation rates in bacteria, as 61.1% of mutators in *Neisseria meningitidis* do not contain mutations in MMR components [58]. This is also exemplified in *Helicobacter pylori*, in which clinical isolates exhibit mutation rates higher than wildtype Enterobacteriaceae and, in 25% of isolates, frequencies higher than MMR-defective mutators of *P. aeruginosa*, yet *H. pylori* lacks MutS-dependent MMR [69]. Despite the lack of *mutS*, *mutL* and *mutH* homologues encoded in the *Mycobacterium tuberculosis* genome, the frequency of spontaneous mutations in this species is similar to other bacteria possessing functional MMR systems [70]. It is possible that the mutator phenotype arising from loss of MMR has been suppressed by additional mutations in this species.

Studies exist which suggest disease epidemics can arise from mutator phenotypes which allows expansion into a new environmental niche [58]. For example, not all isolates of *N. meningitides* are invasive, and 90% of disease has been caused by a limited number of serotypes which differ in antigenic properties of the polysaccharide capsule. Within one of these serotypes exist hypervirulent lineages which are responsible for epidemic outbreaks, and mutators were only observed (57%) in these strains (menA strains) [58]. Disease epidemics can also arise from transient mutator phenotypes which allow better adaptation to an environmental niche but are subsequently reversed to prevent deleterious accumulation of mutations and a subsequent loss of fitness. For example, the W-Beijing *M. tuberculosis* strains, which exhibit a selective advantage over other strains, evidenced by global dissemination and success and several global TB epidemics, and are associated with drug resistance (82% of MDR strains), lack *mutS*, *mutL* and *mutH* in the genome [70]. Despite this, no increase in mutation rate is observed [70]. W-Beijing strains have mutations in other genes which normally prevent mutations caused by oxidative stress, *mutT4* (78%) and *mutT2* (70%), suggesting possible reversion of a mutator phenotype may have occurred [70].

## 4. Mutators Exist within Clinical Populations of Eukaryotic Pathogens

Mutators are also found in clinical populations of eukaryotic pathogens, although the prevalence and clinical significance remains contentious. Many studies have identified fungal clinical isolates with variation in the sequence of the MMR gene *MSH2.* However, these studies did not determine mutation rate, and some alleles were subsequently found not to result in a mutator phenotype [33]. The first study to identify fungal strains with a possible mutator phenotype was in *Candida glabrata*, where 55% of clinical strains recovered from patients were found to possess non-synonymous variation in *MSH2*, and this correlated with anti-fungal drug resistance [32]. In 96% of these *C. glabrata MSH2* variants, the nucleotide sequence variants resulted in amino acid changes in the connector region of the predicted protein which is essential for interaction between the Msh2–Msh3/6 (MutS) and Pms1–Mlh1 (MutL) complexes [32]. However, analysis of mutation rates was not performed due to *C. glabrata* being resistant to canavanine, the drug used in fluctuation assays to determine mutation rate with *S. cerevisiae*, and due to differences in the drug resistance profiles of clinical isolates preventing the use of drug resistance as the reporter [35]. Additional populations of *C. glabrata* clinical isolates were subsequently also shown to have non-synonymous variation in *MSH2* (India, 69%; France, 44%; South Korea, 65%; China, 77%; Spain, 44% and Australia 37%) [71,72,73,74,75,76]. More recently, a novel and improved method was developed for measuring mutation rates in clinical isolates using the green fluorescent protein (GFP) and fluorescence-activated cell sorting (FACS) [33]. This assay showed that there was no difference between mutation rates between two isolates differing in naturally occurring *C. glabrata MSH2* alleles found in the initial study by [32], the type sequence and *MSH2^E231G/L269F^* [33]. This suggests that non-synonymous variation in *MSH2* in clinical isolates does not always result in a mutator phenotype and the prevalence of mutators in clinical populations of *C. glabrata* is overestimated [33,35]. This study highlights the importance of verifying that clinical strains with sequence in variation in *MSH2* have a mutator phenotype before any contribution of *MSH2* mutations to the emergence of anti-fungal drug resistance or clinical outcomes can be evaluated. Another possible complication is allele incompatibility. In *S. cerevisiae*, negative epistatic interactions, which result in a ~100-fold elevation in mutation rate, have been shown to occur between *MLH1* and *PMS1* alleles of the MMR pathway in order to provide an adaptive advantage in high salt stress [77,78,79,80]. Allele incompatibility is proposed to enable a transient increase in mutation rate which can be later eliminated by either mating with non-mutators or through the accumulation of suppressors to avoid any long-term fitness costs [79,80]. It is possible that some of the *MSH2* sequence variants may only exhibit an elevated mutation rate in combination with other specific MMR alleles.

*MSH2* sequence variants have also been identified in a collection of environmental and clinical *Aspergillus fumigatus* isolates at 18.2% frequency and in clinical isolates of the fungal pathogen *Cryptococcus neoformans* [30,31,81,82,83]. Some of these *MSH2* mutants possess a ~200-fold increase in mutation rate compared to wildtype [30]. A recent study on the recurrence of meningitis due to *C. neoformans* also identified an isolate with mutations in the *RAD5* DNA repair gene and the MMR genes *MSH2* and *MSH5* [81]. These mutations lead to a dramatic increase in the number of small nucleotide polymorphisms (SNPs), consistent with the strain being a mutator [81].

It was initially postulated that the outbreak of cryptococcosis caused by *Cryptococcus deuterogattii* that began in the Pacific Northwest of the United States and western Canada in the late 1990s was a result of a transient mutator phenotype [82]. This outbreak is comprised of three clonal expansions which differ in terms of virulence and prevalence [82]. The most virulent isolate of these three clonal expansions, VGIIa, has three closely-related, less-virulent isolates which possess a mutator phenotype and frame-shift mutations in *MSH2* [31,82]. This suggests that a transient mutator phenotype may have played a role in the evolution of virulence in the clonal outbreak [82]. However, by assessing the numbers of indels in homopolymer tracts commonly observed in *MSH2* mutants, Billmyre et al. showed that the VGIIa group did not experience a transient period of *MSH2*-mediated hypermutation, but rather, the mutator phenotype represents an alternative route of adaptation to a new environment [31]. Billmyre et al. also showed that the *MSH2* mutations were not directly responsible for the decrease in virulence observed in the VGIIa-like strains but probably are a result of the accumulation of mutations [31].

In a study of *Plasmodium falciparum* parasites, the causative agent of malaria, a small sub-population within 825 samples from Asia and Africa was identified which displayed high levels of genetic diversity, clinical resistance to the drug artemisinin and sequence variation in the MMR proteins encoded by Pms1, Mlh1 and UvrD [84].

## 5. A Mutator Phenotype Is Associated with Increased Resistance to Antibiotics in Bacteria

A mutator phenotype in bacteria is associated with enhanced evolution of resistance to antibiotics. Mutator strains can acquire resistance more rapidly than non-mutator strains. This is evident in clinical populations in bacterial species such as *Pseudomonas* *aeruginosa*, *Burkholderia cepacia*, *Staphylococcus aureus* and *Salmonella enterica*, in which mutator strains are significantly more resistant to antibiotics than non-mutator strains [49,54,56,57,61,85,86,87]. Serial whole genome sequencing of *P. aeruginosa* isolates from a patient, in whom clonal ceftazidime-avibactam (CZA) resistance emerged over the course of days, showed a lack of MMR which facilitated the rapid evolution of resistance during acute infection [87]. In vitro adaptive evolution experiments with a *mutS* strain also showed rapid acquisition of CZA resistance in vitro [87]. In addition, almost all multi-drug-resistant (MDR) *P. aeruginosa* strains (91.7–100%) and 88.8% of *Haemophilus influenzae* MDR isolates are mutators [54,65,66]. The most frequent modifications observed in multi-drug-resistant (imipenem, ceftazidime, piperacillin, ciprofloxacin and gentamicin) strains were overproduction of the efflux system MexXY-OprM, loss of OprD and overproduction of the β-lactamase AmpC [65]. The higher frequency of mutations leading to resistance has significant relevance to chronic infections which require years of ongoing antibiotic therapy. It has been shown that both the number of mutators and the antibiotic resistance rates among *P. aeruginosa* isolates from chronic infections (e.g., CF patients) are substantially higher than the number of mutators and levels of resistance in other settings such as acute infections in intensive care units [49,52,54]. In a study by Watson et al., six *H. influenzae* mutator isolates of clonal origin were sequentially isolated from a single CF patient over a period of 11 months. This study showed that the MICs for the antibiotics used in treatment increased by up to 4-fold in the last three isolates, thus demonstrating how a natural mutator strain may evolve antibiotic resistance over time [60]. In another study, Ferroni et al. showed by analyzing consecutively isolated strains from the same patients that *P. aeruginosa* mutator strains in a clinical setting are three times quicker in acquiring antibiotic resistance compared to non-mutators [86].

In addition to repairing base mismatches, MMR also prevents recombination between divergent sequences by detecting and removing intermediates that form during recombination between partially homologous sequences [15]. The requirement for sequence homology during recombination is greatly reduced in MMR mutants, enhancing inter-generic recombination [15,88]. MMR mutants increase transductional recombination by 10^2^–10^3^-fold in different serovars of *Salmonella enterica* with low sequence divergence (Typhimurium and Typhi; 1–2%) and also between species with high divergence such as *E.coli* and *Salmonella typhimurium* (~20%) [15,88]. Recombination between divergent species acts as a barrier to horizontal gene transfer, an important mechanism in the acquisition of antibiotic resistance genes in bacteria [8,15,16]. Therefore, MMR mutants are expected to have increased horizontal gene transfer of resistance genes, although there have been no direct studies showing this to date [8].

## 6. The Clinical Association between Mutators and Anti-Microbial Drug Resistance in Eukaryotic Pathogens Remains Controversial

The clinical relevance of mutators in fungal pathogens is strongly contentious and has been compounded by mis-ascribing clinical isolates possessing non-synonymous sequence variation in the MMR gene *MSH2* the term mutators, even though an elevated mutation rate has not been shown. Some clinical isolates with naturally occurring *MSH2* alleles, previously called mutators, do not possess a mutator phenotype [33]. Although non-synonymous variation in *MSH2* was found in *C. glabrata* clinical populations around the world, only some studies have shown a correlation with anti-fungal drug resistance (North America, 65%; South Korea, 69%; China, 43%) [32,74,75]. No correlation was observed between *MSH2* sequence and anti-fungal drug resistance in India, France, Spain and Australia, and in the Australian study the same *MSH2* sequence variants were found in both azole-resistant and -susceptible clinical isolates [71,72,73,76]. The lack of correlation between drug resistance and *MSH2* variants could indicate that a mutator phenotype is advantageous prior to drug exposure but has the added advantage of enabling rapid development of resistance when required [89]. Hypermutation has been found in ancient lineages of fungi which are not pathogenic, suggesting it may have a general advantage in changing environmental conditions [36].

Regardless, mutations in *MSH2* in fungi have been strongly associated with an increase in the generation of spontaneous resistance to numerous clinically relevant anti-fungals in vitro (azoles, polyenes, pyrimidine analogues and echinocandins). Disruption of *msh2* in *C. glabrata* did not alter antibiotic minimum inhibitory concentrations (MICs) but lead to ~82–18- and 9-fold more spontaneously arising caspofungin (echinocandin)-, fluconazole (azole)- and amphotericin B (polyene)-resistant mutants compared to wildtype, in addition to increased spontaneous resistance to micafungin (echinocandin) and voriconazole (azole) [32]. *msh2* deletions also lead to an increased frequency of mutations conferring anti-fungal resistance in an in vivo model of mouse candidiasis, and analysis of serial clinical isolates showed that the *msh2* mutations preceded the emergence of this anti-fungal drug resistance [32]. Resistance to the echinocandin drugs arose in the Δ*msh2* mutant in the primary mechanism of resistance in *C. glabrata*, mutations in *fks1* or *fks2* hotspots (encoding 1,3-ß-D-glucan synthase complex components) [32]. Gain-of-function mutations in the transcription factor encoded by *pdr1*, which lead to overexpression of drug efflux pumps, occurred in 71% of fluconazole-resistant *C. glabrata* Δ*msh2* mutants [32]. A mutation in *erg6* was identified in one amphotericin B-resistant *C. glabrata* Δ*msh2* mutant [32]. Like in *C. glabrata*, deletion of *msh2* in *Candida albicans* and *Cryptococcus neoformans* resulted in an increase in the number of spontaneously arising fluconazole-resistant strains [90,91]. *msh2* mutants in *C. neoformans* also result in an increase in the emergence of resistance to amphotericin B [91]. The *Cryptococcus deuterogattii* Δ*msh2* has been shown to more frequently acquire resistance to 5FC, a drug which is commonly used in combination with amphotericin B [89]. Whole genome sequencing of 5FC-resistant Δ*msh2* mutants revealed mutations in *FCY2* (encodes a permease which transports 5FC into the cell), *FUR1* (encodes a uracil phosporibosyltransferase which, after conversion by cytosine deaminase, processes 5FU to a product which inhibits DNA and protein synthesis) and *UXS1* (encodes an enzyme which converts UDP-glucuronic acid to UDP-xylose) [89]. Mutation of *USX1* leads to the accumulation of UDP-glucuronic acid and altered nucleotide metabolism, which confers 5FC resistance [89]. The *A. fumigatus* Δ*msh2* mutant showed no difference compared to wildtype in the sensitivity to the anti-fungal drugs voriconazole, posaconazole or caspofungin [83]. The Δ*msh2* mutant was subsequently passaged without selection in 10 independent populations and phenotypes of resulting strains analyzed. Compared to the wildtype and original Δ*msh2* mutant, all evolved strains showed no differences in growth, conidiation and sensitivity to DNA damaging agents, but the majority of Δ*msh2* strains became 200-fold more resistant to posaconazole [83].

The role of mutators in the emergence of drug resistance in the malaria parasite *Plasmodium* is complicated by the fact that the *Plasmodium* genome encodes two *msh2* homologs which may have overlapping functions. Only one of these homologs has been successfully disrupted, and this had had no significant effect on drug resistance [92]. However, since then, two strains of *P. falciparum* exhibiting accelerated resistance to multiple drugs and three chloroquine-resistant *P. falciparum* parasites were found to have defective MMR, suggesting that mutators also play a role during the emergence of drug resistance in this eukaryotic pathogen [93]. Lee et al. identified two artemisinin-resistant isolates carrying mutations in two MMR genes, *MLH1* and *PMS1*, which result in a mild mutator phenotype [94].

## 7. Genotypic Modifications in Bacteria Which Facilitate Adaptation to Specific Niches in the Host Environment

Mutation of MMR repair genes enable the rapid accumulation of mutations and genetic variation which may aid in adapting to the host environment. One of the mechanisms of adapting to a host is by altering the surface-exposed molecules that are in direct contact with the host environment. The bacterium *N. meningitides*, a commensal inhabitant of the nasopharynx which can progress to meningococcal disease in rare cases, has evolved a technique which capitalizes on the frequent occurrence of mutations in unstable short DNA repeats. These DNA repeats, specifically homopolymeric tracts, are also one of the most common mutations occurring in MMR mutants. During the process termed phase variation, mutations resulting in reading frame shifts or promoter strength occur in short DNA repeats within or near genes encoding surface-exposed molecules, and this results in fluctuating expression levels [58]. Mutators possess phase variation rates, which are 100-fold higher than wildtype [95]. Using two-phase variation controlled loci *hpuA* and *hmbR*, which contain homopolymeric tract (poly(G) tracts), Richardson et al. showed virulent isolates exhibited significantly longer tracts, and although phase variation frequencies rise linearly with tract length in both wildtype and mutator backgrounds, phase variation frequencies are three orders of magnitude higher in mutators compared to wildtype [58].

Chronic bacterial infections are concomitant with CF. Common pathogens in CF chronic infections include *P. aeruginosa* and *B. cepacia* complex and emerging pathogens such as *Achromobacter xylosoxidans* [96]. In chronic infections of CF patients, *P. aeruginosa* undergoes genetic diversification in the hypoxic and mucoid environment. A higher mutation frequency allows a transition from the virulent variant observed in acute infections to an avirulent variant better adapted to the host environment. Common variants which are correlated with decreased lung function and poor prognosis include quorum-sensing-deficient mucoid and smooth or rough colonies with altered metabolism [97]. During chronic infections, *P. aeruginosa* mutators accumulate 13-fold more mutations per year compared to non-mutators [49]. Long-term persistence of mutators has been observed, indicating that the fitness costs associated with a mutator phenotype are not high enough to impede chronic infection [49]. *mutS* mutants from chronic infections show increased resistance to antibiotics, metabolic adaptations and a loss of a set of established virulence-associated traits [50]. *P. aeruginosa* mutator strains alter their metabolism to adapt to the anaerobic mucus environment by increasing transcription of genes involved in fatty acid and amino acid metabolism, as well as genes involved in the production of energy such as the anaerobic arginine–deiminase pathway, anaerobic respiration, microaerobic respiration, the tricarboxylic acid cycle and glyoxylate shunt [98]. Mutator strains lose virulence-traits essential for acute *P. aeruginosa* infections such as protease activity, pyoverdin production, the Type III secretion system, secreted effectors, antigenicity, motility, osmotic balance, iron acquisition, cytoxicity and quorum sensing [51,99]. Mutators also accumulate mutations which lead to a transition from a non-mucoid to a mucoid growth variant, which is a critical factor for colonization of the respiratory tract in chronic infections [100,101]. Mucoid variants overproduce alginate, a virulence factor which protects the bacterium from the host immune response and which is required for the construction of biofilms. The main target for conversion to mucoidy is the *mucA* gene, which encodes an anti-sigma factor that negatively regulates alginate production [102]. The majority of mucoid isolates (~85%) possess mutations in *mucA* [100,103]. A mutator phenotype, due to mutations in *mutS*, increases the frequency of *mucA* mutations and therefore the frequency of mucoid variants [101]. The most frequent *mucA* mutation in mucoid mutators is the *mucA22* mutation, a deletion of a G residue within a homopolymeric tract [101]. In a separate study of an Argentinean population of CF patients, although 42% of *P. aeruginosa* strains from CF patients were mutators with mutations in *mutS* or *mutL*, no correlation was observed between the mutator phenotype and mutations in *mucA* or either of the other two genes commonly altered in early CF infections, *lasR* (quorum-sensing regulator) and *mexZ* (negative regulator of multi-drug efflux pump) genes [50]. The formation of *P. aeruginosa* biofilms in the CF mucus contributes to higher tolerance to antibiotics and resistance to the immune defense and results in chronic *P. aeruginosa* infection. When grown in structured biofilms, *mutS* mutators have advanced adaptability and increased competitiveness compared to wildtype [97]. Mutators exhibit enhanced micro-colony development and a wider range of architectural features and colony morphology variation which contribute to the ability to cause a chronic infection [97]. *mutS* mutators have also been associated with chronic infections and adaptation to the host environment in the *B. caepacia* complex and emerging CF pathogens such as *A. xylosoxidans* [57,96,104]. Transcriptional analysis of a *mutS* clinical isolate, in addition to other *Burkholderia pseudomallei* isolates from chronic infections, has shown that chronically adapted isolates up-regulate pathways that enhance survival in the host environment such as the quorum-sensing operon, the pyocyanin-insensitive cytochrome *bd* quinal oxidase required for growth in restricted oxygen conditions and resistance–nodulation–division (RND) efflux pump to reduce the effectiveness of antibiotics [105].

The inactivation and deletion of genes is a common feature of host adaption and the development of persistent and chronic infections [106]. An *Enterococcus faecalis* isolate from a patient with recurrent endophthalmitis (inflammation of the interior of the eye) was also shown by whole genome sequencing to carry a mutation in *mutS* [107]. This isolate displayed an increased mutation rate and increased resistance to ceftazidime and carried mutations in genes encoding cell wall-associated proteins, membrane-associated proteins and transcriptional regulators, which may have contributed to drug resistance and the ability to persist and cause a recurrent infection [107]. Sequencing of a *S. enterica* isolate from a chronic infection in an immunocompromised patient over a 15-year period showed that the infection was caused by the same isolate, not re-infection, and that *mutS* lead to the gradual inactivation and loss of genes which were expendable for a systemic lifestyle [106]. Mutator strains also lead to an increase in chronic persistence of *E. coli* urinary tract infections in a mouse model [41].

*mutS* mutations have been shown to provide a temporary adaptive strategy to overcome oxidative stress encountered upon host colonization. Wang et al. (2018) performed whole genome transposon sequencing on mice colonized with *V. cholerae*, the causative agent of cholera, with and without antioxidant treatment. *mutS* mutants displayed increased colonization in mice producing abundant ROS but not those treated with an antioxidant [108]. Increased colonization was a result of the overproduction of catalase and increased biofilm production (a rugose colony morphology), a phenotypic variation which could be readily reversed [108].

Mutators also lead to changes in colony morphology in some species which are associated with adaptation to particular niches. In *Vibrio parahaemolyticus*, a bacterium found in mollusks and sediments in costal environments, mutations in *mutS* increase the frequency of phase variation and the transition from opaque to a translucent colony morphology [68]. Inactivation of *mutS* in *P. aeruginosa* has been shown to spontaneously give rise to diverse colony morphologies [109]. One variant, mS2, is translucent and hyperpigmented and is mainly a result of transition mutations occurring within the *lasR* gene which encodes a quorum-sensing transcriptional activator [109,110]. It is postulated mutations in *lasR* would provide an adaptive advantage by increasing cell viability in the late stationary phase [110]. The association of mutators with mutations in *mucA* and *lasR* highlight how in the first stages of adaptation mutations often occur in global regulators.

## 8. Increased Phenotypic Diversity in Eukaryotic Pathogens Contributes to Host Adaptation

Mutator strains in eukaryotic pathogens result in increased phenotypic diversity and an avenue to adapt to specific environmental niches within the host environment. A mutator strain has been shown increase the frequency of phenotypic switching in *C. neoformans* [111]. Phenotypic switching occurs when cells with a yeast morphology switch to a pseudo-hyphal morphology after in vitro culturing or after ingestion by a slime mold (*Dictyostelium discoideum*) or amoeba (*Acanthamoeba* species) [111,112,113]. Pseudo-hyphal strains can revert back to wildtype yeast morphology at a high frequency both in vitro and in vivo in amoeba or in a murine infection model [111]. A switch to a pseudo-hyphal morphology is hypothesized to prevent phagocytosis by macrophages of the host’s immune system during infection. Pseudo-hyphal strains possess mutations in the RAM signaling pathway (regulation of Ace2p activity and cellular morphogenesis) with *TAO3* being the primary target for mutation [111]. The mutator strain used in these phenotypic switching studies possesses a mutation in the exonuclease proofreading domain of the DNA polymerase delta sub-unit encoded by *POL3* [114]. Similar to *msh2* mutants, a *pol3^D270G^* mutant results in an increased frequency of single nucleotide mutations [30,114]. However, these are predominantly transitions and transversions and not indels at homopolymeric tracts [114]. Reversion of the point mutations within genes of the RAM signaling pathway occurred frequently during murine infection, subsequently reverting the morphology back to yeast [111]. This highlights how a mutator phenotype can allow the frequent loss of function of genes but also provides the means to either adapt to the consequences or directly revert the original mutations to restore function. This is also highlighted in another study with *C. deuterogattii* in which *msh2* mutants displayed frequent phenotypic switching between a red and white colony color and vice versa due to mutations resulting in loss-of-function mutations and reversion of these mutations in genes of the adenine synthesis pathway. Colonies of *ade2* mutants are red due to the accumulation of a red-colored toxic intermediate in vacuoles. Reversion to a white colony color is readily obtained by the generation of suppression mutations to avoid *ade2*-associated toxicity [31]. The *C. deuterogattii msh2* mutant shows frequent generation of red *ade2* colonies and also the frequent reversion back to white colonies [31].

In vitro micro-evolution experiments in *C. neoformans* have revealed that micro-evolution and the generation of phenotypic diversity occurs more rapidly in an Pol3 exonuclease mutant (*pol3*^D270G^) and MMR mutants (Δ*msh2*, Δ*pms1* and Δ*mhl1*) compared to wildtype [30,114]. Phenotypic diversity was observed in traits associated with the ability to grow in vivo including growth at 37 °C, resistance to oxidative stress, alterations in the cell wall and melanization [30]. Interestingly, the Δ*msh2* strain showed more phenotypic diversity than the other strains, possibly indicating why *mutS* and *msh2* mutants are the mostly commonly isolated mutators in clinical populations [30].

The *A. fumigatus* Δ*msh2* mutant and a clinical strain with the A45T and P329T allelic variants exhibited attenuated virulence in both a murine model of invasive aspergillosis and a *Galleria mellonella* infection model. However, 10 independent populations of passaged Δ*msh2* mutants regained virulence, suggesting that the lack of *MSH2* in *A. fumigatus* allows micro-evolution of its virulence attributes in vivo [83].

## 9. Conclusions

Microorganisms rely on mutations to produce novel genetic variation to enable them to survive and adapt to rapidly changing environments. It is becoming apparent that mutators present in clinical populations of pathogenic microbes can enhance adaptive evolution when a population encounters an environment where large-effect beneficial mutations exist to enhance survival. Mutators have been shown to facilitate the emergence of resistance to antibiotics, allow phenotypic modifications to avoid both recognition and destruction by the host immune system and enable switching to cellular morphologies better able to survive in the given environment. Most mutators characterized to date carry mutations in components of the mismatch repair pathway. However, it is clear that there are additional genotypes leading to a mutator phenotype that remain undiscovered in both prokaryotes and eukaryotes. Recent studies have begun to elucidate the genetic factors regulating the phenotypic modifications observed in mutators and highlight an important principle of adaptation, in that in the first stages of adaptation, mutations often occur in global regulators or signaling pathways with pleiotropic effects. Further investigation into these genotypic modifications will greatly enhance our understanding of pathogenesis and adaptation to environmental niches.

## Figures and Tables

**Table 1:** Frequency of Mutator Phenotypes in Clinical Populations of Bacteria.

Species	Number of Mutators	Mutator Frequency	Source of Isolates	Genetic Basis for Mutator ^1^	Reference
*Escherichia coli*	4/212	1.9%	Pathogenic	4/4 *mutS*	[59]
	1/216	0.5%	Commensal	*-*	[63]
	3/288	1.0%	Pathogenic	*-*	[63]
	11/91	12.1%	UTI	-	[64]
*Salmonella* *enterica*	5/137	3.6%	Pathogenic	3/5 *mutS*, 1/5 *mutH*, 1/5 *uvrD*	[59]
*Pseudomonas aeruginosa*	25/128	19.5%	CF patients	6/17 *mutS*, 3/17 *mutY*, 8/17 NI	[49]
	33/62	53.2%	Bronchiectasis and COPD patients	11/17 *mutS*	[54]
	43/79	54%	CF patients	-	[52]
	11/12	92%	CF patients	-	[65]
	3/10	30%	Non-CF patients	-	[65]
*Staphylococcus aureus*	5/124	4%	Clinical isolates	1/4 *mutS*/*mutL* 3/4 *mutL*	[61]
	13/89	14.6%	CF patients	6/13 *mutS* 5/13 NI (not *mutS*)	[56]
	1/74	1.4%	Non-CF patients	-	[56]
*Hemophilus influenzae*	12/500	2.4%	CF patients	7/8 *mutS*	[60]
	18/124	14.5%	CF patients	-	[66]
*Neisseria* *meningitidis*	54/95	56.8%	Invasive clinical isolates	5/54 *mutS* 16/54 *mutL* 33/54 other	[58]
	4/18	22.2%	Meningococcal clinical isolates	1/4 *mutS* 1/4 *mutS*/*mutL* 1/4 *mutL* 1/4 NI	[62]
*Klebsiella pneumoniae*	33/227	14.5%	Clinical isolates	-	[67]
*Vibrio* *parahaemolyticus*	4/78	5.13%	Environmental isolates	-	[68]
	0/64	0%	Clinical isolates	-	[68]
*Stenotrophomonas maltophilia*	17/78	21.8%	CF patients	2/2 *mutS*	[48]

^1^ NI: Not identified.

## Data Availability

No new data were created or analyzed in this study. Data sharing is not applicable to this article.

## References

[1] Taddei F., Radman M., Maynard-Smith J., Toupance B., Gouyon P.H., Godelle B. (1997). Role of mutator alleles in adaptive evolution. Nature.

[2] Giraud A., Matic I., Tenaillon O., Clara A., Radman M., Fons M., Taddei F. (2001). Costs and benefits of high mutation rates: Adaptive evolution of bacteria in the mouse gut. Science.

[3] Tenaillon O., Barrick J.E., Ribeck N., Deatherage D.E., Blanchard J.L., Dasgupta A., Wu G.C., Wielgoss S., Cruveiller S., Medigue C. (2016). Tempo and mode of genome evolution in a 50,000-generation experiment. Nature.

[4] MacLean R.C., Torres-Barcelo C., Moxon R. (2013). Evaluating evolutionary models of stress-induced mutagenesis in bacteria. Nat. Rev. Genet..

[5] Sundin G.W., Weigand M.R. (2007). The microbiology of mutability. FEMS Microbiol. Lett..

[6] Oliver A., Mena A. (2010). Bacterial hypermutation in cystic fibrosis, not only for antibiotic resistance. Clin. Microbiol. Infect..

[7] Miller J.H. (1996). Spontaneous mutators in bacteria: Insights into pathways of mutagenesis and repair. Annu. Rev. Microbiol..

[8] Chopra I., O’Neill A.J., Miller K. (2003). The role of mutators in the emergence of antibiotic-resistant bacteria. Drug Resist. Updates.

[9] Denamur E., Matic I. (2006). Evolution of mutation rates in bacteria. Mol. Microbiol..

[10] Woodford N., Ellington M.J. (2007). The emergence of antibiotic resistance by mutation. Clin. Microbiol. Infect..

[11] Herr A.J., Ogawa M., Lawrence N.A., Williams L.N., Eggington J.M., Singh M., Smith R.A., Preston B.D. (2011). Mutator suppression and escape from replication error-induced extinction in yeast. PLoS Genet..

[12] Fijalkowska I.J., Schaaper R.M. (1996). Mutants in the Exo I motif of *Escherichia coli* dnaQ: Defective proofreading and inviability due to error catastrophe. Proc. Natl. Acad. Sci. USA.

[13] Laengle-Rouault F., Maenhaut-Michel G., Radman M. (1986). GATC sequence and mismatch repair in *Escherichia coli*. EMBO J..

[14] Lahue R.S., Au K.G., Modrich P. (1989). DNA mismatch correction in a defined system. Science.

[15] Rayssiguier C., Thaler D.S., Radman M. (1989). The barrier to recombination between *Escherichia coli* and *Salmonella typhimurium* is disrupted in mismatch-repair mutants. Nature.

[16] Denamur E., Lecointre G., Darlu P., Tenaillon O., Acquaviva C., Sayada C., Sunjevaric I., Rothstein R., Elion J., Taddei F. (2000). Evolutionary implications of the frequent horizontal transfer of mismatch repair genes. Cell.

[17] Saint-Ruf C., Pesut J., Sopta M., Matic I. (2007). Causes and consequences of DNA repair activity modulation during stationary phase in *Escherichia coli*. Crit. Rev. Biochem. Mol. Biol..

[18] Gutierrez A., Laureti L., Crussard S., Abida H., Rodriguez-Rojas A., Blazquez J., Baharoglu Z., Mazel D., Darfeuille F., Vogel J. (2013). beta-Lactam antibiotics promote bacterial mutagenesis via an RpoS-mediated reduction in replication fidelity. Nat. Commun..

[19] Scott J., Thompson-Mayberry P., Lahmamsi S., King C.J., McShan W.M. (2008). Phage-associated mutator phenotype in group A streptococcus. J. Bacteriol..

[20] Chu N.D., Clarke S.A., Timberlake S., Polz M.F., Grossman A.D., Alm E.J. (2017). A mobile element in *mutS* drives hypermutation in a marine *Vibrio*. mBio.

[21] Gerth M., Martinez-Montoya H., Ramirez P., Masson F., Griffin J.S., Aramayo R., Siozios S., Lemaitre B., Mateos M., Hurst G.D.D. (2021). Rapid molecular evolution of *Spiroplasma* symbionts of *Drosophila*. Microb. Genomics.

[22] Dorer M.S., Sessler T.H., Salama N.R. (2011). Recombination and DNA repair in *Helicobacter pylori*. Annu. Rev. Microbiol..

[23] Huang S., Kang J., Blaser M.J. (2006). Antimutator role of the DNA glycosylase *mutY* gene in *Helicobacter pylori*. J. Bacteriol..

[24] Kunkel T.A. (2011). Balancing eukaryotic replication asymmetry with replication fidelity. Curr. Opin. Chem. Biol..

[25] Boiteux S., Jinks-Robertson S. (2013). DNA repair mechanisms and the bypass of DNA damage in *Saccharomyces cerevisiae*. Genetics.

[26] Morrison A., Sugino A. (1994). The 3′-->5′ exonucleases of both DNA polymerases delta and epsilon participate in correcting errors of DNA replication in *Saccharomyces cerevisiae*. Mol. Gen. Genet..

[27] Morrison A., Johnson A.L., Johnston L.H., Sugino A. (1993). Pathway correcting DNA replication errors in *Saccharomyces cerevisiae*. Embo J..

[28] Serero A., Jubin C., Loeillet S., Legoix-Ne P., Nicolas A.G. (2014). Mutational landscape of yeast mutator strains. Proc. Natl. Acad. Sci. USA.

[29] Lang G.I., Parsons L., Gammie A.E. (2013). Mutation rates, spectra, and genome-wide distribution of spontaneous mutations in mismatch repair deficient yeast. G3.

[30] Boyce K.J., Wang Y., Verma S., Shakya V.P.S., Xue C., Idnurm A. (2017). Mismatch repair of DNA replication errors contributes to microevolution in the pathogenic fungus *Cryptococcus neoformans*. MBio.

[31] Billmyre R.B., Clancey S.A., Heitman J. (2017). Natural mismatch repair mutations mediate phenotypic diversity and drug resistance in *Cryptococcus deuterogattii*. eLife.

[32] Healey K.R., Zhao Y., Perez W.B., Lockhart S.R., Sobel J.D., Farmakiotis D., Kontoyiannis D.P., Sanglard D., Taj-Aldeen S.J., Alexander B.D. (2016). Prevalent mutator genotype identified in fungal pathogen *Candida glabrata* promotes multi-drug resistance. Nat. Commun..

[33] Shor E., Schuyler J., Perlin D.S. (2019). A novel, drug resistance-independent, fluorescence-based approach to measure mutation rates in microbial pathogens. MBio.

[34] Tran H.T., Gordenin D.A., Resnick M.A. (1999). The 3′-->5′ exonucleases of DNA polymerases delta and epsilon and the 5′-->3′ exonuclease Exo1 have major roles in postreplication mutation avoidance in *Saccharomyces cerevisiae*. Mol. Cell. Biol..

[35] Boyce K.J., Idnurm A. (2019). Lighting up mutation: A new unbiased system for the measurement of microbial mutation rates. MBio.

[36] Steenwyk J.L., Opulente D.A., Kominek J., Shen X.X., Zhou X., Labella A.L., Bradley N.P., Eichman B.F., Cadez N., Libkind D. (2019). Extensive loss of cell-cycle and DNA repair genes in an ancient lineage of bipolar budding yeasts. PLoS Biol..

[37] Milo S., Harari-Misgav R., Hazkani-Covo E., Covo S. (2019). Limited DNA repair gene repertoire in ascomycete yeast revealed by comparative genomics. Genome Biol. Evol..

[38] Phillips M.A., Steenwyk J.L., Shen X.X., Rokas A. (2021). Examination of gene loss in the dna mismatch repair pathway and its mutational consequences in a fungal phylum. Genome Biol. Evol..

[39] Sniegowski P.D., Gerrish P.J., Lenski R.E. (1997). Evolution of high mutation rates in experimental populations of *E. coli*. Nature.

[40] Tenaillon O., Toupance B., Le Nagard H., Taddei F., Godelle B. (1999). Mutators, population size, adaptive landscape and the adaptation of asexual populations of bacteria. Genetics.

[41] Labat F., Pradillon O., Garry L., Peuchmaur M., Fantin B., Denamur E. (2005). Mutator phenotype confers advantage in *Escherichia coli* chronic urinary tract infection pathogenesis. FEMS Immunol. Med. Microbiol..

[42] Maddamsetti R., Grant N.A. (2020). Divergent Evolution of Mutation Rates and Biases in the Long-Term Evolution Experiment with Escherichia coli. Genome Biol. Evol..

[43] Mao E.F., Lane L., Lee J., Miller J.H. (1997). Proliferation of mutators in a cell population. J. Bacteriol..

[44] Funchain P., Yeung A., Stewart J.L., Lin R., Slupska M.M., Miller J.H. (2000). The consequences of growth of a mutator strain of *Escherichia coli* as measured by loss of function among multiple gene targets and loss of fitness. Genetics.

[45] Zahrt T.C., Buchmeier N., Maloy S. (1999). Effect of *mutS* and *recD* mutations on *Salmonella* virulence. Infect. Immun..

[46] Picard B., Duriez P., Gouriou S., Matic I., Denamur E., Taddei F. (2001). Mutator natural *Escherichia coli* isolates have an unusual virulence phenotype. Infect. Immun..

[47] Kang M., Kim K., Choe D., Cho S., Kim S.C., Palsson B., Cho B.K. (2019). Inactivation of a mismatch-repair system diversifies genotypic landscape of *Escherichia coli* during adaptive laboratory evolution. Front. Microbiol..

[48] Turrientes M.C., Baquero M.R., Sanchez M.B., Valdezate S., Escudero E., Berg G., Canton R., Baquero F., Galan J.C., Martinez J.L. (2010). Polymorphic mutation frequencies of clinical and environmental *Stenotrophomonas maltophilia* populations. Appl. Environ. Microbiol..

[49] Oliver A., Canton R., Campo P., Baquero F., Blazquez J. (2000). High frequency of hypermutable *Pseudomonas aeruginosa* in cystic fibrosis lung infection. Science.

[50] Feliziani S., Lujan A.M., Moyano A.J., Sola C., Bocco J.L., Montanaro P., Canigia L.F., Argarana C.E., Smania A.M. (2010). Mucoidy, quorum sensing, mismatch repair and antibiotic resistance in *Pseudomonas aeruginosa* from cystic fibrosis chronic airways infections. PLoS ONE.

[51] Hogardt M., Hoboth C., Schmoldt S., Henke C., Bader L., Heesemann J. (2007). Stage-specific adaptation of hypermutable *Pseudomonas aeruginosa* isolates during chronic pulmonary infection in patients with cystic fibrosis. J. Infect. Dis..

[52] Ciofu O., Riis B., Pressler T., Poulsen H.E., Hoiby N. (2005). Occurrence of hypermutable *Pseudomonas aeruginosa* in cystic fibrosis patients is associated with the oxidative stress caused by chronic lung inflammation. Antimicrob. Agents Chemother..

[53] Gutierrez O., Juan C., Perez J.L., Oliver A. (2004). Lack of association between hypermutation and antibiotic resistance development in *Pseudomonas aeruginosa* isolates from intensive care unit patients. Antimicrob. Agents Chemother..

[54] Macia M.D., Blanquer D., Togores B., Sauleda J., Perez J.L., Oliver A. (2005). Hypermutation is a key factor in development of multiple-antimicrobial resistance in *Pseudomonas aeruginosa* strains causing chronic lung infections. Antimicrob. Agents Chemother..

[55] Martinez-Solano L., Macia M.D., Fajardo A., Oliver A., Martinez J.L. (2008). Chronic *Pseudomonas aeruginosa* infection in chronic obstructive pulmonary disease. Clin. Infect. Dis..

[56] Prunier A.L., Malbruny B., Laurans M., Brouard J., Duhamel J.F., Leclercq R. (2003). High rate of macrolide resistance in *Staphylococcus aureus* strains from patients with cystic fibrosis reveals high proportions of hypermutable strains. J. Infect. Dis..

[57] Martina P., Feliziani S., Juan C., Bettiol M., Gatti B., Yantorno O., Smania A.M., Oliver A., Bosch A. (2014). Hypermutation in *Burkholderia cepacia* complex is mediated by DNA mismatch repair inactivation and is highly prevalent in cystic fibrosis chronic respiratory infection. Int. J. Med. Microbiol..

[58] Richardson A.R., Yu Z., Popovic T., Stojiljkovic I. (2002). Mutator clones of *Neisseria meningitidis* in epidemic serogroup A disease. Proc. Natl. Acad. Sci. USA.

[59] LeClerc J.E., Li B., Payne W.L., Cebula T.A. (1996). High mutation frequencies among *Escherichia coli* and *Salmonella* pathogens. Science.

[60] Watson M.E., Burns J.L., Smith A.L. (2004). Hypermutable *Haemophilus influenzae* with mutations in *mutS* are found in cystic fibrosis sputum. Microbiology.

[61] Trong H.N., Prunier A.L., Leclercq R. (2005). Hypermutable and fluoroquinolone-resistant clinical isolates of *Staphylococcus aureus*. Antimicrob. Agents Chemother..

[62] Colicchio R., Pagliarulo C., Lamberti F., Vigliotta G., Bruni C.B., Alifano P., Salvatore P. (2006). RecB-dependent mutator phenotype in *Neisseria meningitidis* strains naturally defective in mismatch repair. DNA Repair.

[63] Matic I., Radman M., Taddei F., Picard B., Doit C., Bingen E., Denamur E., Elion J. (1997). Highly variable mutation rates in commensal and pathogenic *Escherichia coli*. Science.

[64] Denamur E., Bonacorsi S., Giraud A., Duriez P., Hilali F., Amorin C., Bingen E., Andremont A., Picard B., Taddei F. (2002). High frequency of mutator strains among human uropathogenic *Escherichia coli* isolates. J. Bacteriol..

[65] Henrichfreise B., Wiegand I., Pfister W., Wiedemann B. (2007). Resistance mechanisms of multiresistant *Pseudomonas aeruginosa* strains from Germany and correlation with hypermutation. Antimicrob. Agents Chemother..

[66] Roman F., Canton R., Perez-Vazquez M., Baquero F., Campos J. (2004). Dynamics of long-term colonization of respiratory tract by *Haemophilus influenzae* in cystic fibrosis patients shows a marked increase in hypermutable strains. J. Clin. Microbiol..

[67] De Champs C., Rich C., Chandezon P., Chanal C., Sirot D., Forestier C. (2004). Factors associated with antimicrobial resistance among clinical isolates of *Klebsiella pneumoniae*: 1-year survey in a French university hospital. Eur. J. Clin. Microbiol. Infect. Dis..

[68] Hazen T.H., Kennedy K.D., Chen S., Yi S.V., Sobecky P.A. (2009). Inactivation of mismatch repair increases the diversity of *Vibrio parahaemolyticus*. Environ. Microbiol..

[69] Bjorkholm B., Sjolund M., Falk P.G., Berg O.G., Engstrand L., Andersson D.I. (2001). Mutation frequency and biological cost of antibiotic resistance in *Helicobacter pylori*. Proc. Natl. Acad. Sci. USA.

[70] Ebrahimi-Rad M., Bifani P., Martin C., Kremer K., Samper S., Rauzier J., Kreiswirth B., Blazquez J., Jouan M., van Soolingen D. (2003). Mutations in putative mutator genes of *Mycobacterium tuberculosis* strains of the W-Beijing family. Emerg. Infect. Dis..

[71] Biswas C., Marcelino V.R., Van Hal S., Halliday C., Martinez E., Wang Q., Kidd S., Kennedy K., Marriott D., Morrissey C.O. (2018). Whole genome sequencing of australian *Candida glabrata* isolates reveals genetic diversity and novel sequence types. Front. Microbiol..

[72] Singh A., Healey K.R., Yadav P., Upadhyaya G., Sachdeva N., Sarma S., Kumar A., Tarai B., Perlin D.S., Chowdhary A. (2018). Absence of azole or echinocandin resistance in *Candida glabrata* isolates in India despite background prevalence of strains with defects in the dna mismatch repair pathway. Antimicrob. Agents Chemother..

[73] Delliere S., Healey K., Gits-Muselli M., Carrara B., Barbaro A., Guigue N., Lecefel C., Touratier S., Desnos-Ollivier M., Perlin D.S. (2016). Fluconazole and echinocandin resistance of *Candida glabrata* correlates better with antifungal drug exposure rather than with *msh2* mutator genotype in a french cohort of patients harboring low rates of resistance. Front. Microbiol..

[74] Byun S.A., Won E.J., Kim M.N., Lee W.G., Lee K., Lee H.S., Uh Y., Healey K.R., Perlin D.S., Choi M.J. (2018). Multilocus Sequence Typing (MLST) genotypes of *Candida glabrata* bloodstream isolates in korea: Association with antifungal resistance, mutations in mismatch repair gene (*msh2*), and clinical outcomes. Front. Microbiol..

[75] Hou X., Xiao M., Wang H., Yu S.Y., Zhang G., Zhao Y., Xu Y.C. (2018). Profiling of *PDR1* and *MSH2* in *Candida glabrata* bloodstream isolates from a multicenter study in China. Antimicrob. Agents Chemother..

[76] Bordallo-Cardona M.A., Agnelli C., Gomez-Nunez A., Sanchez-Carrillo C., Bouza E., Munoz P., Escribano P., Guinea J. (2019). *MSH2* gene point mutations are not antifungal resistance markers in *Candida glabrata*. Antimicrob. Agents Chemother..

[77] Heck J.A., Argueso J.L., Gemici Z., Reeves R.G., Bernard A., Aquadro C.F., Alani E. (2006). Negative epistasis between natural variants of the *Saccharomyces cerevisiae MLH1* and *PMS1* genes results in a defect in mismatch repair. Proc. Natl. Acad. Sci. USA.

[78] Bui D.T., Dine E., Anderson J.B., Aquadro C.F., Alani E.E. (2015). A genetic incompatibility accelerates adaptation in yeast. PLoS Genet..

[79] Raghavan V., Bui D.T., Al-Sweel N., Friedrich A., Schacherer J., Aquadro C.F., Alani E. (2018). Incompatibilities in mismatch repair genes *MLH1-PMS1* contribute to a wide range of mutation rates in human isolates of baker’s yeast. Genetics.

[80] Bui D.T., Friedrich A., Al-Sweel N., Liti G., Schacherer J., Aquadro C.F., Alani E. (2017). Mismatch repair incompatibilities in diverse yeast populations. Genetics.

[81] Rhodes J., Beale M.A., Vanhove M., Jarvis J.N., Kannambath S., Simpson J.A., Ryan A., Meintjes G., Harrison T.S., Fisher M.C. (2017). A population genomics approach to assessing the genetic basis of within-host microevolution underlying recurrent cryptococcal meningitis infection. G3.

[82] Billmyre R.B., Croll D., Li W., Mieczkowski P., Carter D.A., Cuomo C.A., Kronstad J.W., Heitman J. (2014). Highly recombinant VGII *Cryptococcus gattii* population develops clonal outbreak clusters through both sexual macroevolution and asexual microevolution. mBio.

[83] Dos Reis T.F., Silva L.P., de Castro P.A., do Carmo R.A., Marini M.M., da Silveira J.F., Ferreira B.H., Rodrigues F., Lind A.L., Rokas A. (2019). The *Aspergillus fumigatus* mismatch repair *MSH2* homolog is important for virulence and azole resistance. mSphere.

[84] Miotto O., Almagro-Garcia J., Manske M., Macinnis B., Campino S., Rockett K.A., Amaratunga C., Lim P., Suon S., Sreng S. (2013). Multiple populations of artemisinin-resistant *Plasmodium falciparum* in Cambodia. Nat. Genet..

[85] Levy D.D., Sharma B., Cebula T.A. (2004). Single-nucleotide polymorphism mutation spectra and resistance to quinolones in *Salmonella enterica* serovar Enteritidis with a mutator phenotype. Antimicrob. Agents Chemother..

[86] Ferroni A., Guillemot D., Moumile K., Bernede C., Le Bourgeois M., Waernessyckle S., Descamps P., Sermet-Gaudelus I., Lenoir G., Berche P. (2009). Effect of mutator *P. aeruginosa* on antibiotic resistance acquisition and respiratory function in cystic fibrosis. Pediatr. Pulmonol..

[87] Khil P.P., Dulanto Chiang A., Ho J., Youn J.H., Lemon J.K., Gea-Banacloche J., Frank K.M., Parta M., Bonomo R.A., Dekker J.P. (2019). Dynamic emergence of mismatch repair deficiency facilitates rapid evolution of ceftazidime-avibactam resistance in *Pseudomonas aeruginosa* acute infection. mBio.

[88] Zahrt T.C., Maloy S. (1997). Barriers to recombination between closely related bacteria: MutS and RecBCD inhibit recombination between *Salmonella typhimurium* and *Salmonella typhi*. Proc. Natl. Acad. Sci. USA.

[89] Billmyre R.B., Applen Clancey S., Li L.X., Doering T.L., Heitman J. (2020). 5-fluorocytosine resistance is associated with hypermutation and alterations in capsule biosynthesis in *Cryptococcus*. Nat. Commun..

[90] Legrand M., Chan C.L., Jauert P.A., Kirkpatrick D.T. (2008). Analysis of base excision and nucleotide excision repair in *Candida albicans*. Microbiology.

[91] Boyce K.J., Andrianopoulos A. (2011). Ste20-related kinases: Effectors of signaling and morphogenesis in fungi. Trends Microbiol..

[92] Bethke L., Thomas S., Walker K., Lakhia R., Rangarajan R., Wirth D. (2007). The role of DNA mismatch repair in generating genetic diversity and drug resistance in malaria parasites. Mol. Biochem. Parasitol..

[93] Castellini M.A., Buguliskis J.S., Casta L.J., Butz C.E., Clark A.B., Kunkel T.A., Taraschi T.F. (2011). Malaria drug resistance is associated with defective DNA mismatch repair. Mol. Biochem. Parasitol..

[94] Lee A.H., Fidock D.A. (2016). Evidence of a mild mutator phenotype in Cambodian *Plasmodium falciparum* malaria parasites. PLoS ONE.

[95] Richardson A.R., Stojiljkovic I. (2001). Mismatch repair and the regulation of phase variation in *Neisseria meningitidis*. Mol. Microbiol..

[96] Veschetti L., Sandri A., Johansen H.K., Lleo M.M., Malerba G. (2020). Hypermutation as an evolutionary mechanism for *Achromobacter xylosoxidans* in cystic fibrosis lung infection. Pathogens.

[97] Lujan A.M., Macia M.D., Yang L., Molin S., Oliver A., Smania A.M. (2011). Evolution and adaptation in *Pseudomonas aeruginosa* biofilms driven by mismatch repair system-deficient mutators. PLoS ONE.

[98] Hoboth C., Hoffmann R., Eichner A., Henke C., Schmoldt S., Imhof A., Heesemann J., Hogardt M. (2009). Dynamics of adaptive microevolution of hypermutable *Pseudomonas aeruginosa* during chronic pulmonary infection in patients with cystic fibrosis. J. Infect. Dis..

[99] Smith E.E., Buckley D.G., Wu Z., Saenphimmachak C., Hoffman L.R., D’Argenio D.A., Miller S.I., Ramsey B.W., Speert D.P., Moskowitz S.M. (2006). Genetic adaptation by *Pseudomonas aeruginosa* to the airways of cystic fibrosis patients. Proc. Natl. Acad. Sci. USA.

[100] Mathee K., Ciofu O., Sternberg C., Lindum P.W., Campbell J.I., Jensen P., Johnsen A.H., Givskov M., Ohman D.E., Molin S. (1999). Mucoid conversion of *Pseudomonas aeruginosa* by hydrogen peroxide: A mechanism for virulence activation in the cystic fibrosis lung. Microbiology.

[101] Moyano A.J., Lujan A.M., Argarana C.E., Smania A.M. (2007). MutS deficiency and activity of the error-prone DNA polymerase IV are crucial for determining *mucA* as the main target for mucoid conversion in *Pseudomonas aeruginosa*. Mol. Microbiol..

[102] Martin D.W., Schurr M.J., Mudd M.H., Govan J.R., Holloway B.W., Deretic V. (1993). Mechanism of conversion to mucoidy in *Pseudomonas aeruginosa* infecting cystic fibrosis patients. Proc. Natl. Acad. Sci. USA.

[103] Boucher J.C., Yu H., Mudd M.H., Deretic V. (1997). Mucoid *Pseudomonas aeruginosa* in cystic fibrosis: Characterization of *muc* mutations in clinical isolates and analysis of clearance in a mouse model of respiratory infection. Infect. Immun..

[104] Ridderberg W., Jensen Handberg K., Norskov-Lauritsen N. (2020). Prevalence of hypermutator isolates of *Achromobacter* spp. from cystic fibrosis patients. Int. J. Med. Microbiol..

[105] Price E.P., Viberg L.T., Kidd T.J., Bell S.C., Currie B.J., Sarovich D.S. (2018). Transcriptomic analysis of longitudinal *Burkholderia pseudomallei* infecting the cystic fibrosis lung. Microb. Genomics.

[106] Klemm E.J., Gkrania-Klotsas E., Hadfield J., Forbester J.L., Harris S.R., Hale C., Heath J.N., Wileman T., Clare S., Kane L. (2016). Emergence of host-adapted *Salmonella Enteritidis* through rapid evolution in an immunocompromised host. Nat. Microbiol..

[107] Chilambi G.S., Nordstrom H.R., Evans D.R., Kowalski R.P., Dhaliwal D.K., Jhanji V., Shanks R.M.Q., Van Tyne D. (2021). Genomic and phenotypic diversity of *Enterococcus faecalis* isolated from endophthalmitis. PLoS ONE.

[108] Wang H., Xing X., Wang J., Pang B., Liu M., Larios-Valencia J., Liu T., Liu G., Xie S., Hao G. (2018). Hypermutation-induced in vivo oxidative stress resistance enhances *Vibrio cholerae* host adaptation. PLoS Pathog..

[109] Smania A.M., Segura I., Pezza R.J., Becerra C., Albesa I., Argarana C.E. (2004). Emergence of phenotypic variants upon mismatch repair disruption in *Pseudomonas aeruginosa*. Microbiology.

[110] Lujan A.M., Moyano A.J., Segura I., Argarana C.E., Smania A.M. (2007). Quorum-sensing-deficient (*lasR*) mutants emerge at high frequency from a *Pseudomonas aeruginosa mutS* strain. Microbiology.

[111] Magditch D.A., Liu T.B., Xue C., Idnurm A. (2012). DNA mutations mediate microevolution between host-adapted forms of the pathogenic fungus *Cryptococcus neoformans*. PLoS Pathog..

[112] Neilson J.B., Ivey M.H., Bulmer G.S. (1978). *Cryptococcus neoformans*: Pseudohyphal forms surviving culture with Acanthamoeba polyphaga. Infect. Immun..

[113] Steenbergen J.N., Nosanchuk J.D., Malliaris S.D., Casadevall A. (2003). *Cryptococcus neoformans* virulence is enhanced after growth in the genetically malleable host *Dictyostelium discoideum*. Infect. Immun..

[114] Boyce K.J., Wang Y., Xue C., Idnurm A. (2020). A spontaneous mutation in DNA polymerase *POL3* during *in vitro* passaging causes a hypermutator phenotype in *Cryptococcus* species. DNA Repair.

